# Recurrent Hand, Foot, and Mouth Disease in a Saudi Girl

**DOI:** 10.7759/cureus.51813

**Published:** 2024-01-07

**Authors:** Lamia Alakrash, Maee Barakeh, Wasan I AlQahtani, Renad K AlKanaan

**Affiliations:** 1 Department of Dermatology, King Fahad Medical City, Riaydh, SAU; 2 College of Medicine, King Saud University, Riyadh, SAU; 3 College of Medicine, Princess Nourah Bint Abdulrahman University, Riyadh, SAU; 4 College of Medicine, King Saud Medical City, Riyadh, SAU

**Keywords:** viral illness, saudi arabia, pediatric dermatology, children, rash, recurrence, hand-foot-mouth disease

## Abstract

Hand, foot, and mouth disease (HFMD) is a viral illness that predominantly affects infants and children, causing blisters and sores on the hands, feet, and mouth. Recurrence is rare, but a case in a six-year-old girl in Saudi Arabia was reported.

A six-year-old girl presented with a rash on her palms and soles, which was preceded by a mild sore throat and low-grade fever. She had been in contact with her two-year-old sister, who had similar symptoms but a different rash pattern.

During clinical examination, multiple erythematous deep-seated vesicles and papules were noted on the patient's palms and soles, with no involvement of mucous membranes or nails. The diagnosis of hand, foot, and mouth disease (HFMD) was made based on the characteristic clinical presentation, and the rash resolved within seven days without treatment or complications.

The patient had experienced a similar presentation six months ago, which was also diagnosed as HFMD, and the rash had resolved spontaneously within one week. In her second episode, the rash was less severe, with milder prodromal symptoms. In both episodes, the lesions were asymptomatic and had no mucosal involvement. The patient had experienced onychomadesis after her first episode, but no nail abnormalities were seen after her second episode.

Although HFMD is rare to recur in children, outbreaks can lead to another episode. HFMD prevalence is underestimated in Saudi Arabia due to missed mild cases. Pediatricians and dermatologists should be aware of HFMD incidence and its complications, as early detection is vital in preventing outbreaks and transmission.

## Introduction

Hand, foot, and mouth disease (HFMD) is a viral illness that affects people of all ages, but it is most common in infants and children younger than five years old. The infection typically involves the hands, feet, mouth, and occasionally even the genitalia and buttocks [1]. The Coxsackievirus A type 16 and Enterovirus A71 are the most common causes of HFMD. However, many other types of coxsackieviruses and enteroviruses can also cause the disease [1,2]. The recurrence or persistence of HFMD is rare. Due to its rarity, here we are reporting a case of HFMD recurrence in a six-year-old Saudi girl.

## Case presentation

**Figure 1:**
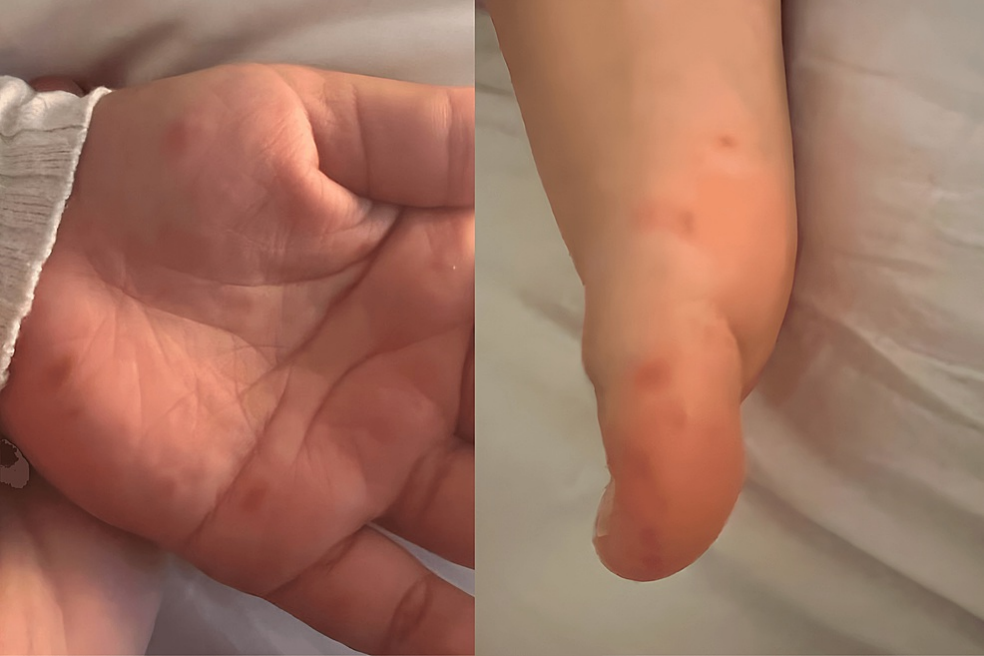
The first episode showed multiple erythematous deep-seated vesicles and papules distributed over palms and soles

**Figure 2:**
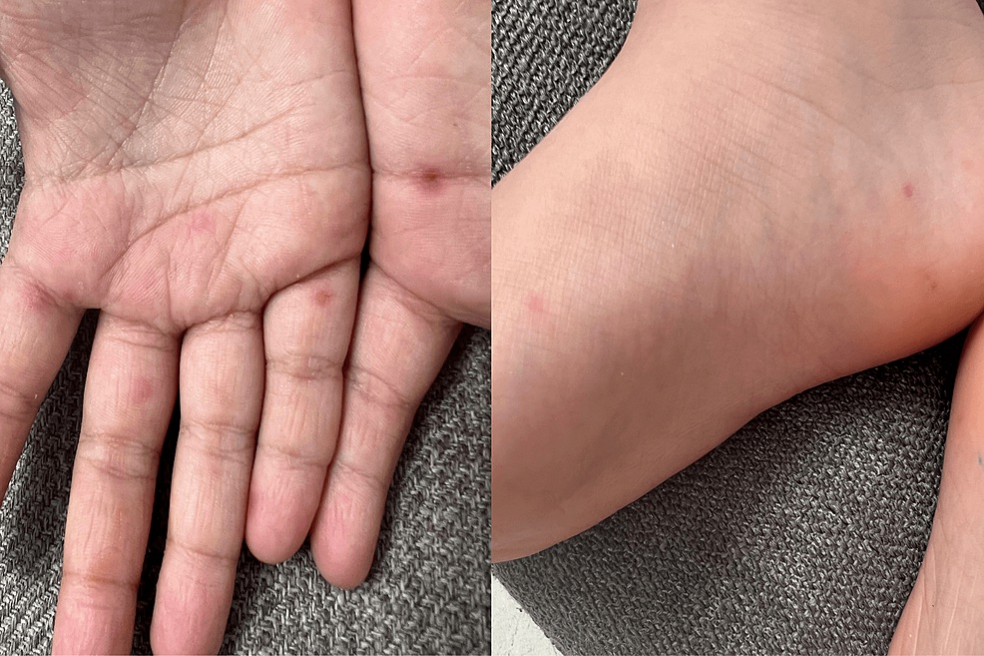
The recurrent episode showed a less severe rash following the same distribution of the previous episode

A six-year-old Saudi girl, not known to have any medical illnesses, presented with a history of asymptomatic rash over bilateral palms and soles for two days, sparing the oral mucosa. The rash was preceded by a mild sore throat and low-grade fever that lasted for one day. Additionally, she has a history of contact with her two-year-old sister, who had similar symptoms, but the rash was confined to the oral mucosa with a few scattered lesions on the buttock area, sparing the palms and soles. The clinical examination of the patient revealed multiple erythematous deep-seated vesicles and papules distributed over palms and soles (Figure 1). The mucous membranes and nails were spared, and no other areas were involved. Due to limitations in polymerase chain reaction (PCR) tests for certain viruses related to HFMD in our facility, the diagnosis of HFMD was made clinically as the patient exhibited a clear and consistent set of clinical symptoms and signs highly suggestive of the disease. The fever was effectively managed with oral ibuprofen, and the rash resolved spontaneously within seven days without necessitating any treatment as it was asymptomatic, with no reported complications. However, the patient gave a history of a similar presentation six months ago. The diagnosis of HFMD was made at that time, and the lesions resolved spontaneously within a week. The rash was less severe in the second episode, with milder prodromal symptoms (Figure 2). Lesions were asymptomatic in both episodes without mucosal involvement, and no treatment was required. She had a history of post-HFMD onychomadesis after the first episode. However, no nail abnormalities were seen after the second episode.

## Discussion

Hand, foot, and mouth disease (HFMD) is a common viral infection, primarily affecting young children under the age of five [3], although it may be rarely seen in adults [4]. It is known to appear in a characteristic distribution involving mucocutaneous lesions over hands, feet, and oral cavity [1].

The etiology of HFMD is attributed to numerous enterovirus strains, with the Coxsackie virus being the predominant viral agent often associated with this condition. Specifically, Coxsackievirus A16 and Enterovirus A71 are by far the most common causes of HFMD outbreaks. After the initial outbreak in California in 1969, the substantial occurrence of HFMD in the Asia-Pacific region and other parts of the world has led to an increased burden among this age group [5]. Many cases were reported about the complications and mortality associated with HFMD. In severe cases, it may lead to serious complications such as central nervous system (CNS) complications like meningitis and encephalitis, cardiopulmonary complications like heart failure and pulmonary edema, or even death. Neurological and cardiopulmonary consequences were the leading causes of mortality, especially in outbreaks [6-8]. The severity stages of severe HFMD can be categorized into three distinct phases: individuals with CNS involvement, those experiencing autonomic nervous system (ANS) dysregulation, and subsequently, those facing evident cardiopulmonary failure, which may encompass conditions such as pulmonary edema or hemorrhage. In response to these concerns, the World Health Organization (WHO) published a guide in 2011 for the clinical management and public health response to HFMD. The management guidelines are based on the initial assessment of HFMD severity. If the patient presents with warning signs of CNS involvement, admission to the pediatric ward or ICU should be considered. However, if the patient presents with skin rash and oral ulcers without warning signs, the patient may be sent home with supportive management, which includes administering paracetamol or ibuprofen and ensuring adequate fluid intake. There is no specific antiviral agent for the etiologic agents. Also, parents should be educated to watch out for warning signs [9].

HFMD is classically a one-time infection in a lifetime, though some cases were reported worldwide for infection recurrence. One theory that explains the recurrence is infection by a different strain with an absence of immunity against it [10]. Multiple studies were conducted during the HFMD outbreak in China. One study estimated the recurrence rate to be 0.44% among patients infected by HFMD from 2008 to 2015. According to the author, 70% of relapsed patients aged from two to five years old [11]. 

Our case would be the first case reported of a recurrent HFMD in Saudi Arabia. The explanation of recurrence may be a new genus of viral infection, decrease in immunity, or concomitant asymptomatic COVID-19 infection, but herein, an idiopathic insult was recorded. In the case presented, the presentation of the recurrence was milder compared to the first episode as the lesions were less severe without nail involvement, and the prodromal symptoms were milder. This presentation is contradictory to other reported cases, where the second episode was more severe, and lesions were more painful [12,13].

Our presented case, along with other reported cases, manifested the same lesion distribution in both the first and second episodes. In addition, a complete resolution of symptoms without subsequent complications was reported [12-14].

## Conclusions

Recurrence of HFMD in children is a rare phenomenon but not unusual, mostly reported in outbreaks. Documentation of recurrent HFMD in Saudi Arabia is lacking, and disease prevalence is underestimated. This can be attributed to HFMD cases being often overlooked or not diagnosed properly in pediatric clinics, as well as to instances of mild illness that resolve without requiring a hospital visit. The rising incidence of cases worldwide necessitates heightened awareness among pediatricians and dermatologists. Early disease identification is crucial to minimize the risk of transmission to others and prevent potential outbreaks.
